# Colon cancer modulation by a diabetic environment: A single institutional experience

**DOI:** 10.1371/journal.pone.0172300

**Published:** 2017-03-02

**Authors:** Isabel Prieto, Laura del Puerto-Nevado, Nieves Gonzalez, Sergio Portal-Nuñez, Sandra Zazo, Marta Corton, Pablo Minguez, Carmen Gomez-Guerrero, Jose Miguel Arce, Ana Belen Sanz, Sebastian Mas, Oscar Aguilera, Gloria Alvarez-Llamas, Pedro Esbrit, Alberto Ortiz, Carmen Ayuso, Jesus Egido, Federico Rojo, Jesus Garcia-Foncillas, on behalf of the DiabetesCancerConnect Consortium

**Affiliations:** 1 Radiation Oncology, Oncohealth Institute, IIS-Fundacion Jimenez Diaz- UAM, Madrid, Spain; 2 Translational Oncology Division, Oncohealth Institute, IIS-Fundacion Jimenez Diaz-UAM, Madrid, Spain; 3 Renal, Vascular and Diabetes Research Laboratory, IIS-Fundacion Jimenez Diaz-UAM, Madrid, Spain; 4 Spanish Biomedical Research Network in Diabetes and Associated Metabolic Disorders (CIBERDEM), Madrid, Spain; 5 Bone and Mineral Metabolism Laboratory, IIS-Fundacion Jimenez Diaz-UAM, Madrid, Spain; 6 Pathology Department, IIS-Fundacion Jimenez Diaz-UAM, Madrid, Spain; 7 Department of Genetics, IIS-Fundacion Jimenez Diaz-UAM, Madrid, Spain; 8 Health Information Management Department, Fundacion Jimenez Diaz, Madrid. Spain; 9 Immunoallergy and proteomics Laboratory, IIS-Fundacion Jimenez Diaz-UAM, Madrid, Spain; Baylor University Medical Center, UNITED STATES

## Abstract

**Background:**

Multiple observational studies suggest an increased risk of colon cancer in patients with diabetes *mellitus* (DM). This can theoretically be the result of an influence of the diabetic environment on carcinogenesis or the tumor biologic behavior.

**Aim:**

To gain insight into the influence of a diabetic environment on colon cancer characteristics and outcomes.

**Material and methods:**

Retrospective analysis of clinical records in an academic tertiary care hospital with detailed analysis of 81 diabetic patients diagnosed of colon cancer matched with 79 non-diabetic colon cancer patients. The impact of streptozotocin-induced diabetes on the growth of colon cancer xenografts was studied in mice.

**Results:**

The incidence of DM in 1,137 patients with colorectal cancer was 16%. The diabetic colon cancer cases and non-diabetic colon cancer controls were well matched for demographic and clinical variables. The ECOG Scale Performance Status was higher (worse) in diabetics (ECOG ≥1, 29.1% of controls vs 46.9% of diabetics, p = 0.02), but no significant differences were observed in tumor grade, adjuvant therapy, tumor site, lymphovascular invasion, stage, recurrence, death or cancer-related death. Moreover, no differences in tumor variables were observed between patients treated or not with metformin. In the xenograft model, tumor growth and histopathological characteristics did not differ between diabetic and nondiabetic animals.

**Conclusion:**

Our findings point towards a mild or negligible effect of the diabetes environment on colon cancer behavior, once cancer has already developed.

## Background

Diabetes *mellitus* and cancer are among the most frequent causes of death worldwide [1–3]. Epidemiologic evidence suggests an association between diabetes *mellitus* and an increased risk of many forms of cancer [4–6]. Colon cancer is the third -in men- and the second -in women- most common cancer in the global population [2]. The results of multiple observational studies, but not all, have suggested that diabetes may significantly increase the risk of colon cancer [7–11]. However, data from specific studies have been disputed as based on heterogeneous groups, with unreliable ascertainment of diabetes, and using sources with poorly rigorous data such as Surveillance, Epidemiology, and End Results, which may have led to inconclusive or misleading results [9, 12–14]. Accordingly, the magnitude of the association may have been potentially influenced by hints and bias [15, 16]. It also remains uncertain whether the putative link between diabetes and colon cancer is direct, related to hyperglycemia; whether diabetes is an indicator of latent biologic factors that modify cancer risk such as insulin resistance and hyperinsulinemia; or whether the diabetes-cancer association is indirect and reflects the influence of common risk factors such as age, lifestyle behaviors, overweight, poor dietary habits or therapeutic agents [17]. Moreover, there is a limited knowledge related to the influence of a diabetic environment on the carcinogenesis process, the biologic behavior of the tumor, the response to different treatments and patient outcomes [5, 18]. *In vitro* and *in vivo* studies have supported a direct role of high glucose concentrations in tumor development and characteristics [19]. In this respect, animal models have addressed the influence of diabetes/insulin resistance on carcinogenesis (e.g. carcinogenic molecules used in Leptin receptor–deficient db/db mice) [20], or of diabetes/hyperglycemia on tumor xenograft progression (e.g. glucagon-induced hyperglycemia [21] or streptozotocin-induced diabetes in mice [22, 23]), although colon cancer had not been previously studied in depth.

We have now aimed at gaining insight into the influence of a diabetic environment on colon cancer characteristics and progression through a combination of clinical studies and a preclinical evaluation of the influence of the hyperglycemia on tumor xenograft progression.

## Material and methods

### Clinical study

The study is a retrospective patient record analysis carried out at Fundacion Jimenez Diaz Hospital, for patients from January 2009 to December 2013. Clinical data and biopsies were obtained after informed consent from patients underwent surgery for purposes not related to this study. Candidate patients were identified using the specific institutional software named Alcor (Sigesa, 2014). This program works with an encoded system, which matched the specific requested terms. The first research setting included the variables colorectal cancer and diabetes, and the second diabetes, cancer and colorectal cancer.

#### Study sample

From the total number of patients found with a diagnosis of colorectal cancer and also diabetes at the institution, only 81 diabetic patients met the inclusion criteria established for this study:

Patients with resection of primary colon cancer, histological type colon adenocarcinoma, colon location (rectal cancer patients were excluded), time from surgery up to 6 months, no neoadjuvant treatment, no other concurrent neoplasia or immunosuppressive treatment, and diabetes diagnosed as a documented registry of diabetes, or historical anti-diabetic medication intake or meeting the American Diabetic Association (ADA) criteria for diabetes at time of reviewing the data. The ADA criteria used to determine if patients had diabetes were: Hemoglobin A1c values ≥ 6.5%, or fasting blood glucose levels ≥ 125 mg/dL, with high fasting values recorded 2 or more times or random blood glucose levels ≥ 200 mg/dL, with high random values recorded 2 or more times (S1 Fig).

In parallel, this observational study also included 79 non-diabetic patients with primary diagnosis of colon cancer, who underwent resection during the same period, using equal inclusion criteria except the presence of diabetes, aiming to obtain a well-balanced series.

Baseline variables recorded for all patients were age, gender, metformin intake, clinical debut (acute or subacute symptoms), Eastern Cooperative Oncology Group (ECOG) Scale Performance Status, Body Mass Index (BMI), adjuvant therapy, overall survival, disease free survival, and cause of death if deceased. Other variables recorded from peripheral blood at diagnosis were glucose, triglycerides, cholesterol, white blood cell levels, and carcinoembryonic antigen (CEA) level.

Tumor characteristics were analyzed based on the following criteria:

- Information regarding depth of invasion or thickness of the tumor (pT) was collected from the pathology reports after surgery, as defined by the American Joint Committee on Cancer Criteria for colon and rectal cancer staging (https://cancerstaging.org/About/Pages/8th-Edition.aspx).
- Tumors were graded as low grade (G1-G2), and high grade (G3) following the 2010 WHO classification (http://www.pathologyoutlines.com/topic/colontumorwhoclassification.html).
- Tumor location was categorized as proximal or right (cecum, hepatic flexure, ascending and transverse colon), and distal or left (splenic flexure and descending colon) [24].
- Information regarding lymphovascular invasion was collected from the pathology report, determined on hematoxylin and eosin staining. Vein invasion was identified as present if tumor cells were observed in an endothelial-lined channel with a smooth muscle wall. Lymphatic vessel invasion was identified as present if tumor cells were observed in an endothelial-lined channel devoid of smooth muscle.
- TNM staging: Data on Classification of Malignant Tumours (TNM) staging was collected from the clinical registries in the computerized system and was assessed based on pathology reports available after surgical resection and results from imaging studies. The information was coded according to the TNM staging described in the American Joint Committee on Cancer (AJCC) eighth edition (https://cancerstaging.org/Pages/default.aspx). For analytical purposes, T stages were classified as low T stage (T1-T2) or high T stage (T3-T4), N stages were considered as N0 (absence of node involvement) and N+ (lymph node metastases) and final stage as low stage (0, I, any II) or high stage (any III, IV).

The study was approved by the Institutional Scientific and Ethical Committee at IIS-Fundacion Jimenez Diaz (Madrid, Spain) (CEIC-FJD, approval code 08/13; on October 1st, 2013) in accordance with the ethical principles stated in the Declaration of Helsinki. Informed consent is included in the clinical history of each participant and recorded by the standard requirements of data protection rules established by the SPANISH DATA PROTECTION AGENCY (LOPD 15/1999).

### Tumor xenograft model in streptozotocin-induced diabetic mice

Fifteen male athymic mice NU-Foxn1nu (8-week-old) were purchased from Charles River Laboratories and housed in the specific pathogen-free room of the Animal Model Core Facility of Research Health Institute–Fundacion Jimenez Diaz (ES28079000089). Mice were maintained in individually ventilated cages (IVCs) with a 12:12 h light-dark cycle. 2014 Teklad global 14% protein irradiated diet and autoclaved water was provided *ad libitum*.

All animal procedures and experimental protocols were approved by the Ethical Animal Research Committee at IIS-Fundacion Jimenez Diaz (Madrid, Spain) and were also conducted in accordance with institutional standards (Reference n°: PROEXP 024–15), which fulfilled the requirements established by the Spanish government and the European Community (Real Decreto R.D. 53/2003).

#### Diabetes induction

Diabetes was chemically induced by a single intraperitoneal injection of 200 mg/Kg body weight streptozotocin (STZ, Sigma-Aldrich) in a total volume of 200 μl in 50 mM citrate buffer (pH = 4.5) in 10 animals. The control group (5 mice) received 200 μl citrate buffer. Ten days after the STZ administration, 60% of STZ injected animals presented blood glucose above 200 mg/dl and were considered as the streptozotocin-induced diabetic (STZ-D) group. Blood glucose levels were monitored periodically during the experiment, but animals did not receive insulin or other antidiabetic drug.

#### Xenograft implantation

**Fig 1:**
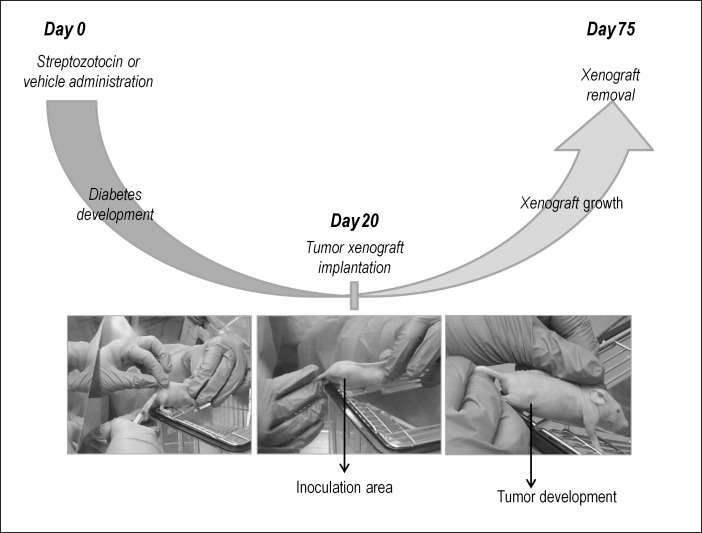
Schematic representation of the cancer xenograft model in mice.

The colorectal cancer HT29 cell line, recently classified as “metabolic subtype” [25, 26], was used to generate a xenograft twenty days after streptozotocin or vehicle administration. Cells were cultured in RPMI-1640 medium (Gibco) with 10% fetal bovine serum (FBS) at 37°C in a 5% CO_2_ atmosphere. Medium was supplemented with penicillin G (100 U/ml), and streptomycin (0.1 mg/ml). A volume of 200 μl containing 2x10^6^ cells [1:1 mixture of PBS: Matrigel (BD Biosciences)] was injected subcutaneously into both flanks of the animal. The size of the tumor was measured three times a week using a Vernier caliper along two perpendicular axes and volume was calculated with the formula: volume = (length × width^2^)/2 (Fig 1).

#### Pathological assessment

Fifty-five days after tumor induction, mice were sacrificed by using carbon dioxide (CO2) euthanasia and tumors were removed and fixed in 10% neutral buffered formalin solution for 24 hours and embedded in paraffin wax. All samples were processed following the same procedure. Tissues were cut into 4-μm-thick sections, and stained with hematoxylin-eosin stain for morphological examination by light microscopy.

CD31-vascular structures in tumor were also evaluated by immunohistochemistry. Consecutive 4-μm tissue sections were obtained from formalin-fixed paraffin-embedded samples. Antigen retrieval was performed in PT-Link (Dako) for 20 min at 95°C in high pH buffered solution (Dako). Endogenous peroxidase was blocked by immersing the sections in 0.03% hydrogen peroxide for 5 min. Slides were washed for 5 min with Tris buffered saline solution containing Tween 20 at pH 7.6 and incubated with a primary antibody for CD31 (dilution 1:25, Clone JC70A, Abcam) for 20 min at room temperature, followed by incubation with the appropriate anti-Ig horseradish peroxidase-conjugated polymer (EnVision, Dako). Sections were then visualized with 3, 3’-diaminobenzidine as a chromogen for 5 min and counterstained with hematoxylin. All stainings were performed in an Autostainer platform (Dako). CD31-vascular structures were counted in ten ×200 magnification microscopic fields from the area of highest vascular density in the tumor.

### Statistical analysis

Data were analyzed by SPSS v.20.0 software. Continuous variables were expressed as mean ± standard deviation, and categorical variables were expressed as frequencies and percentages. Demographic, clinical characteristics, and tumor pathologic variables were compared between patients with and without diabetes by chi-square or Fisher´s exact test when appropriate, in case of categorical variables and by t-test in case of continuous variables. A *p* value lower than 0.05 was considered statistically significant, in all analyses.

## Results

### Epidemiological data

**Fig 2:**
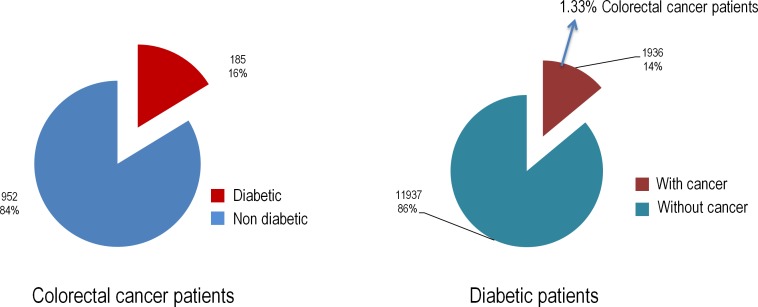
Overview of the stratified data, provided by the medical records of patients at Fundacion Jimenez Diaz.

Among 1,137 patients diagnosed of incident colorectal cancer from January 2009 to December 2013 found, diabetes was present in 185 (16%); the incidence of any cancer in among 13,873 diabetic patients was 14%, being 1.3% of these cases colorectal cancer (Fig 2).

### Colon cancer characteristics in diabetic versus non-diabetic patients

Descriptive and clinical results and frequencies of variables of diabetic and non-diabetic colon cancer patients that met inclusion criteria are presented in Table 1. Demographic and clinical characteristics in both arms demonstrated the homogeneity of the group. The mean age was 72.9 (range, 45–95 years) for non-diabetic patients and 76.7 (range, 46–91) for diabetic patients.

**Table 1:** Clinicopathological characteristics of non-diabetic and diabetic colon cancer patients. Data expressed as mean ± SD or N (%).

*Variable*	*Overall (N = 160)*	*Non-diabetic (N = 79)*	*Diabetic (N = 81)*	P
***Age*** years,	74.8 ± 10.4	72.9 ± 11.3	76.7 ± 9.2	0.02
***Glucose levels***, mg/dl,	112.4 ± 38.5	93.2 ± 9.0	131.1 ± 46.4	<0.0001*
***Triglycerides*,** mg/dl,	117.8 ±71.0	95.4 ±36.1	138.0 ± 87.3	<0.0001*
***Cholesterol*,** mg/dl,	165.8 ± 44.2	180.3 ±38.9	151.1 ±40.3	<0.0001*
***BMI*,** kg/m^2^,	24.4 ±2.8	23.5 ± 2.7	25.9 ±2.5	0.06
***Lymphocytes***, x10^3^ μl,	2.1 ±0. 9	2.1 ± 0.8	2.1 ± 0.9	0.70
***Neutrophils***, x10^3^ μl,	4.7 ± 2.1	4.3 ± 1.9	5.0 ± 2.1	0.02*
***Platelets***, x10^3^ μl,	283.0 ± 116.0	279.2 ± 111.8	286.1 ± 120.1	0.70
**Females**, N (%)	67 (41.9%)	37 (46.8%)	30 (37%)	0.21
***Clinical debut colon cancer***, N (%)				
Subclinical	146 (91.2%)	74 (93.7%)	72 (88.9%)	0.28
Acute	14 (8.8%)	5 (6.3%)	9 (11.1%)	
***ECOG***, N (%)				
0	99 (61.9%)	56 (70.9%)	43 (53.1%)	0.02*
≥ 1	61 (38.1%)	23 (29.1%)	38 (46.9%)	
***CEA (ng/mL)***, N (%)				
≤5	87 (54.4%)	47 (82.5%)	40 (75.5%)	0.39
>5	23 (14.4%)	10 (17.5%)	13 (24.5%)	
N/A	50 (31.2%)	-	-	
***pT*,** N (%)				
T1-T2	53 (33.1%)	24 (30.4%)	29 (35.8%)	0.61
T3-T4	107 (66.9%)	55 (69.6%)	52 (64.2%)	
***pN*,** N (%)				
N0	101 (63.1%)	48 (60.8%)	53 (65.4%)	0.54
N+	59 (36.9%)	31 (39.2%)	28 (34.6%)	
***Grade***, N (%)				
Low grade	147 (91.9%)	72 (91.1%)	75 (92.6%)	0.73
High grade	13 (8.1%)	7 (8.9%)	6 (7.4%)	
***Adjuvant therapy***, N (%)	54 (33.8%)	29 (36.7%)	25 (30.9%)	0.43
***Tumor site***, N (%)				
Right	75 (46.9%)	32 (40.5%)	43 (53.1%)	0.11
Left	85 (53.1%)	47 (59.5%)	38 (46.9%)	
***Lymphovascular invasion*,** N (%)				
Yes	23 (14.4%)	12 (18.8%)	11 (16.4%)	0.72
No	108 (67.5%)	52 (81.2%)	56 (83.6%)	
N/A	29 (18.1%)	-	-	
***Stage*,** N (%)				
Low	100 (62.5%)	49 (62.0%)	51 (63.0%)	0.90
High	60 (37.5%)	30 (38.0%)	30 (37.0%)	
***Recurrence***, N (%)	16 (10.0%)	7 (8.9%)	9 (11.1%)	0.63
***Death*,** N (%)	23 (14.4%)	11 (13.9%)	12 (14.8%)	0.87
***Cancer-related death*,** N (%**)	12 (52.1%)	5 (45.4%)	7 (58.3%)	0.53

* p<0.05 denotes statistical significance.

** % of total deaths.

Abbreviations: BMI, Body mass index; ECOG, Eastern Cooperative Oncology Group; CEA, carcinoembrionary antigen; SD, standard deviation; N/A, Not available

Results were homogeneous with respect to gender, clinical debut and CEA levels, while serum glucose, triglycerides and circulating neutrophils were significantly higher and serum cholesterol significantly lower in diabetics and there was a non-significant trend towards higher BMI in diabetics.

ECOG Scale Performance Status was 0 in 99 patients (61.9%), and ≥1 in the rest of patients (38.1%); with the diabetic group having higher prevalence of high scores (ECOG ≥1 29.1% in non-diabetics *vs* 46.9% in diabetics, p = 0.02).

Regarding tumor variables, no significant differences were found between diabetics and non diabetics in the prevalence of high T stage, node involvement, grade of differentiation, presence of lymphovascular invasion, tumor location, recurrence rate, death events or cancer-related deaths.

From the total of diabetic patients, 35 were treated with metformin. No significant differences were observed in tumor-related features between diabetic patients on metformin or not on metformin (Table 2).

**Table 2:** Cancer-related clinicopathological characteristics and metformin use in diabetic patients with colon cancer.

*Variable*	*Non Metformin (N = 46)*	*Metformin(N = 35)*	*P*
***ECOG*,** *N(%)*			
0	27 (58.7%)	16 (45.7%)	0.24
≥ 1	19 (41.3%)	19 (54.3%)	
***pT*,** *N(%)*			
T1-T2	18 (39.1%)	11 (31.4%)	0.47
T3-T4	28 (60.8%)	24 (68.5%)	
***pN*,** *N(%)*			
N0	31 (67.4%)	22 (62.9%)	0.67
N+	15 (32.6%)	13 (37.1%)	
***Grade*,** *N(%)*			
Low grade	42 (91.3%)	33 (94.3%)	0.69
High grade	4 (8.7%)	2 (5.7%)	
***Lymphovascular invasion*,** *N(%)*			
Yes	7 (15.2%)	4 (11.4%)	0.75
No	32 (69.6%)	24 (68.6%)	
N/A	7(15.2%)	7 (20%)	
***Stage*,** *N(%)*			
Low	30 (65.2%)	21 (60.0%)	0.63
High	16 (34.8%)	14 (40.0%)	
***Recurrence*,** *N(%)*			
Yes	6 (13.0%)	3 (8.6%)	0.75
No	40 (87.0%)	32 (91.4%)	
***Death*,** *N(%)*	7 (15.2%)	5 (14.3%)	0.90
***Cancer-related death*,** N(%*)	4 (57.1%)	3 (60%)	0.29

* % of total deaths

Abbreviations: ECOG, Eastern Cooperative Oncology Group; N/A, Not available

### A diabetic environment does not modify tumor xenograft growth in STZ-D mice

Since clinical data did not support a relationship between diabetes and specific tumor features, the influence of diabetes on the growth and histological features of human colon cancer xenografts was assessed in mice, following the experimental protocol represented in Fig 1.

**Fig 3:**
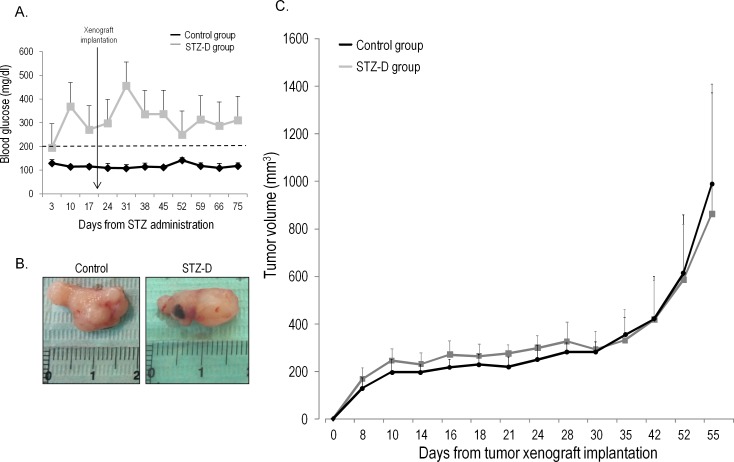
A diabetic environment does not modify tumor xenograft growth in mice. **A)** Blood glucose levels in diabetic and control mice during the experiment. The cutoff point to define development of diabetes was established at 200 mg/dl; **B)** Representative images of tumors from diabetic and control mice; **C)** Tumor growth curves for xenografts in diabetic and control mice.

STZ-D mice displayed mean levels of blood glucose >200 mg/dl through the experiment (Fig 3A). At the end of follow-up no significant differences in tumor volume were observed between control and STZ-D mice (Fig 3B and 3C).

**Fig 4:**
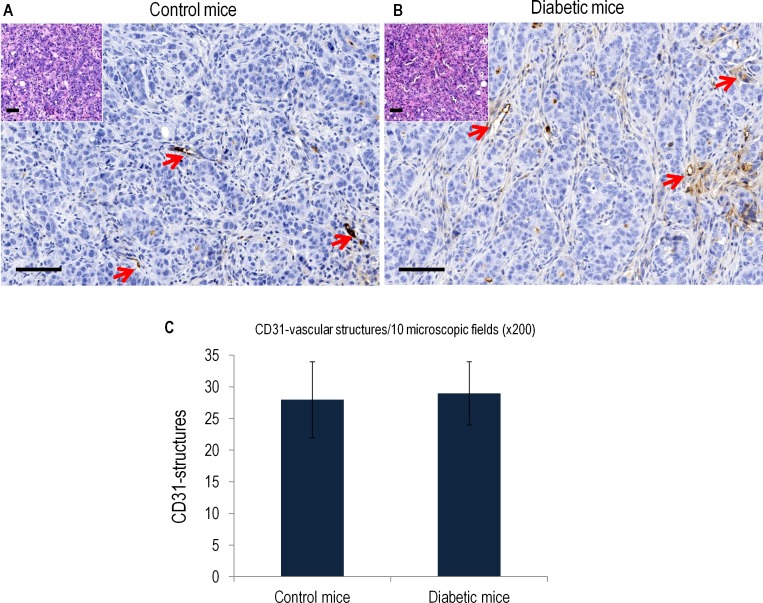
Representative histological images and corresponding quantification of vascular structures in the tumor xenograft model in mice. **A) and B)** show hematoxylin-eosin staining and CD31 immunohistochemistry images in tumor samples from control and diabetic xenografts. Original magnification x200. Arrows indicate vascular structures. **C)** Graphical representation of the number of vascular structures/10 microscopic x200 fields in control and diabetic mice. Scale, bar, 100 μm.

The morphological analysis of tumors in control and STZ-D groups revealed identical architectural and cytologic characteristics, showing a homogeneous solid growth pattern with scattered differentiated glands, invasive tumor edge on surrounding soft tissues and focal intratumoral necrosis. Similarly, no differences in proliferation rates were observed (Fig 4A and 4B). The examination of other organs did not demonstrated dissemination of tumor cells.

Analysis of microvascular density in tumors using CD31 as endothelial marker for quantification also demonstrated similar results between control and STZ-D mice (Fig 4C).

## Discussion

There are many epidemiological studies addressing the risk of developing cancer in diabetic patients, but few reports have focused on the influence of diabetes on colon cancer behavior once colon cancer has already developed. We now report that no differences in tumor behavior or characteristics were found when colon cancer in diabetic patients was compared with colon cancer in non-diabetic patients or when colon cancer xenografts were implanted in diabetic or control mice.

The main strength of the present study is the homogeneous, well-balanced and well-characterized population of the clinical study that limits the bias that may result from more heterogeneous or less characterized populations [12, 14, 27, 28]. Another strength is a preclinical study that supports the conclusions of the clinical dataset.

Our clinical results are in accordance to those in the “European American” ethnic group, ethnically closer to our cohort, in a multiethnic cohort [12]. Thus, there were no differences between diabetics and controls in terms of anatomical or pathological characteristics as stage or right/left localization. A previous report describing an association between diabetes and worse histopathologic colorectal cancer features may have been limited by excessive patient heterogeneity related to the inclusion of diverse histological types and of patients with rectal cancer [28]. Colon and rectal cancer show multiple biological and clinical differences, probably related to different mechanism of oncogenesis [24, 29, 30]. Furthermore, the standard treatment for rectal cancer shows more inter-institutional variability depending on the surgeon expertise. We excluded rectal cancer from our study in order to better preserve the anatomical structure. Standard rectal cancer treatment includes neoadjuvant chemo-radiation, which essentially modifies cell integrity and the microenvironment and may make the tumor disappear. Thus, rectal cancer patients merit a separate analysis. Adenocarcinoma is the most common colon cancer subtype and the most probably linked with a pro-inflammatory environment. Thus, we excluded other histologic types, since additional oncogenic mechanisms may contribute.

It has been suggested that medication used to control the glucose levels, mainly metformin, may modify the risk of developing cancer [31–34]. For this reason, we analyzed the metformin-treated subgroup in our cohort. We did not observe an effect of metformin on tumor behavior. However, we are very cautious about this result, since the additional stratification of data resulted in very low number of cases. Nevertheless, despite the few cases analyzed, results were concordant with several reports, which showed that metformin may reduce cancer risk, but its effect on mortality following cancer remains unclear [35, 36], even in other tumor types [37].

The preclinical *in vivo* model is based on published and widely accepted methods [38], yielding an homogeneous and reproducible experimental approach which allows for non-biased robust results. This experimental procedure disclosed no significant differences in tumor growth kinetics or histopathological characteristics between diabetic and control mice. Very few *in vivo* studies have addressed the influence of diabetes on the growth of human cancer. One report did not observe significant differences in sarcoma xenograft growth kinetics between diabetic and control mice [23]. By contrast, in a similar experimental setting, a protective effect of diabetes in a prostate cancer xenograft model was reported [22]. However, the published patient cohort reports are heterogeneous regarding to the effect of diabetes on prostate cancer patients [39].

Among the weaknesses, we should mention the retrospective nature of the study and the relatively small study population, belonging to a single center. While the smaller size allowed a degree of detail in the analysis that is not possible in large epidemiological studies, the single center nature limits the extrapolation of results to other centers or countries.

To summarize, our findings point towards a mild or negligible influence of diabetes on colon cancer behavior once cancer has already developed. These results serve as the basis to design a larger, multicenter, confirmatory clinical study.

## Supporting information

S1 Fig Schematic representation of the selection of patients who met the inclusion criteria for database development. (TIF)

## Abbreviations

ADA: American Diabetic Association
ECOG: Eastern Cooperative Oncology Group
BMI: Body Max Index
pT: Invasion depth or thickness of the tumor
AJCC: American Joint Committee on Cancer
STZ: streptozotocin
CEA: carcinoembryonic antigen
FBS: Fetal Bovine Serum

## References

[1] International Diabetes Federation. IDF Diabetes Atlas. 7th ed. Brussels, Belgium; 2015.

[2] Lozano R, Naghavi M, Foreman K, Lim S, Shibuya K, Aboyans V, et al Global and regional mortality from 235 causes of death for 20 age groups in 1990 and 2010: a systematic analysis for the Global Burden of Disease Study 2010. Lancet. 2012;380: 2095–128. 10.1016/S0140-6736(12)61728-0 23245604 PMC10790329

[3] Siegel R, Ma J, Zou Z, Jemal A. Cancer statistics. CA Cancer J Clin. 2014;64: 9–29. 10.3322/caac.21208 24399786

[4] Johnson JA, Carstensen B, Witte D, Bowker SL, Lipscombe L, Renehan AG; Diabetes and Cancer Research Consortium. Diabetes and cancer (1): evaluating the temporal relationship between type 2 diabetes and cancer incidence. Diabetologia. 2012;55: 1607–18. 10.1007/s00125-012-2525-1 22476947

[5] Renehan AG, Yeh HC, Johnson JA, Wild SH, Gale EA, Møller H; Diabetes and Cancer Research Consortium. Diabetes and cancer (2): evaluating the impact of diabetes on mortality in patients with cancer. Diabetologia. 2012;55: 1619–32. 10.1007/s00125-012-2526-0 22476948

[6] Suh S, Kim KW. Diabetes and cancer: is diabetes causally related to cancer? Diabetes Metab J. 2011;35: 193–8. 10.4093/dmj.2011.35.3.193 21785737 PMC3138100

[7] Dankner R, Boffetta P, Balicer RD, Boker LK, Sadeh M, Berlin A, et al Time-Dependent Risk of Cancer After a Diabetes Diagnosis in a Cohort of 2.3 Million Adults. Am J Epidemiol. 2016;183: 1098–106. 10.1093/aje/kwv290 27257115

[8] de Kort S, Simons CC, van den Brandt PA, Goldbohm RA, Arts IC, de Bruine AP, et al Diabetes mellitus type 2 and subsite-specific colorectal cancer risk in men and women: results from the Netherlands Cohort Study on diet and cancer. Eur J Gastroenterol Hepatol. 2016;28: 896–903. 10.1097/MEG.0000000000000626 27097356

[9] Harding JL, Shaw JE, Peeters A, Cartensen B, Magliano DJ. Cancer risk among people with type 1 and type 2 diabetes: disentangling true associations, detection bias, and reverse causation. Diabetes Care. 2015;38: 264–70. 10.2337/dc14-1996 25488912

[10] Jarvandi S, Davidson NO, Schootman M. Increased risk of colorectal cancer in type 2 diabetes is independent of diet quality. PLoS One. 2013;8: e74616 10.1371/journal.pone.0074616 24069323 PMC3771921

[11] Wang M, Hu RY, Wu HB, Pan J, Gong WW, Guo LH, et al Cancer risk among patients with type 2 diabetes mellitus: a population-based prospective study in China. Sci Rep. 2015;5: 11503 10.1038/srep11503 26082067 PMC4469976

[12] He J, Stram DO, Kolonel LN, Henderson BE, Le Marchand L, Haiman CA. The association of diabetes with colorectal cancer risk: the Multiethnic Cohort. Br J Cancer. 2010;103: 120–6. 10.1038/sj.bjc.6605721 20531412 PMC2905298

[13] Wang JY, Chao TT, Lai CC, Wang CY, Wu VC, Wang SM, et al Risk of colorectal cancer in type 2 diabetic patients: a population-based cohort study. Jpn J Clin Oncol. 2013;43: 258–63. 10.1093/jjco/hys228 23288931

[14] Jiang Y, Ben Q, Shen H, Lu W, Zhang Y, Zhu J. Diabetes mellitus and incidence and mortality of colorectal cancer: a systematic review and meta-analysis of cohort studies. Eur J Epidemiol. 2011;26: 863–76. 10.1007/s10654-011-9617-y 21938478

[15] Cogliano V, Straif K. Re: False-positive results in cancer epidemiology: a plea for epistemological modesty. J Natl Cancer Inst. 2010;102: 134; author reply 134–5. 10.1093/jnci/djp446 20007526

[16] De Bruijn KM, Ruiter R, de Keyser CE, Hofman A, Stricker BH, van Eijck CH. Detection bias may be the main cause of increased cancer incidence among diabetics: results from the Rotterdam Study. Eur J Cancer. 2014;50: 2449–55. 10.1016/j.ejca.2014.06.019 25047425

[17] Giovannucci E, Harlan DM, Archer MC, Bergenstal RM, Gapstur SM, Habel LA, et al Diabetes and cancer: a consensus report. Diabetes Care. 2010;33: 1674–85. 10.2337/dc10-0666 20587728 PMC2890380

[18] Vissers PA, Falzon L, van de Poll-Franse LV, Pouwer F, Thong MS. The impact of having both cancer and diabetes on patient-reported outcomes: a systematic review and directions for future research. J Cancer Surviv. 2016;10: 406–15. 10.1007/s11764-015-0486-3 26428396 PMC4801990

[19] Masur K, Vetter C, Hinz A, Tomas N, Henrich H, Niggemann B, et al Diabetogenic glucose and insulin concentrations modulate transcriptome and protein levels involved in tumour cell migration, adhesion and proliferation. Br J Cancer. 2011;104: 345–52. 10.1038/sj.bjc.6606050 21179032 PMC3031898

[20] Algamas-Dimantov A, Yehuda-Shnaidman E, Hertz R, Peri I, Bar-Tana J, Schwartz B. Prevention of diabetes-promoted colorectal cancer by (n-3) polyunsaturated fatty acids and (n-3) PUFA mimetic. Oncotarget. 2014;5: 9851–63. 10.18632/oncotarget.2453 25375205 PMC4259442

[21] Wang Y, Zhu YD, Gui Q, Wang XD, Zhu YX.Glucagon-induced angiogenesis and tumor growth through the HIF-1-VEGF-dependent pathway in hyperglycemic nude mice. Genet Mol Res. 2014;13: 7173–83. 10.4238/2014.September.5.3 25222223

[22] Barbosa-Desongles A, Hernández C, De Torres I, Munell F, Poupon MF, Simó R, et al Diabetes protects from prostate cancer by downregulating androgen receptor: new insights from LNCaP cells and PAC120 mouse model. PLoS One. 2013;8: e74179 10.1371/journal.pone.0074179 24058525 PMC3769234

[23] da Silva Faria MC, Santos NA, Carvalho Rodrigues MA, Rodrigues JL, Barbosa Junior F, Santos AC. Effect of diabetes on biodistribution, nephrotoxicity and antitumor activity of cisplatin in mice. Chem Biol Interact. 2015;229: 119–31. 10.1016/j.cbi.2015.01.027 25665769

[24] Li FY, Lai MD. Colorectal cancer, one entity or three. J Zhejiang Univ Sci B, 2009;10: 219–29. 10.1631/jzus.B0820273 19283877 PMC2650032

[25] Guinney J, Dienstmann R, Wang X, de Reyniès A, Schlicker A, Soneson C, et al The consensus molecular subtypes of colorectal cancer. Nat Med. 2015;21: 1350–6. 10.1038/nm.3967 26457759 PMC4636487

[26] Sadanandam A, Lyssiotis CA, Homicsko K, Collisson EA, Gibb WJ, Wullschleger S, et al A colorectal cancer classification system that associates cellular phenotype and responses to therapy. Nat Med. 2013;19: 619–25. 10.1038/nm.3175 23584089 PMC3774607

[27] Deng L, Gui Z, Zhao L, Wang J, Shen L. Diabetes mellitus and the incidence of colorectal cancer: an updated systematic review and meta-analysis. Dig Dis Sci. 2012;57: 1576–85. 10.1007/s10620-012-2055-1 22350783

[28] Sharma A, Ng H, Kumar A, Teli K, Randhawa J, Record J, et al, Colorectal cancer: Histopathologic differences in tumor characteristics between patients with and without diabetes. Clin Colorectal Cancer. 2014;13: 54–61. 10.1016/j.clcc.2013.10.002 24342823

[29] Kapiteijn E, Liefers GJ, Los LC, Kranenbarg EK, Hermans J, Tollenaar RA, et al Mechanisms of oncogenesis in colon versus rectal cancer. J Pathol. 2001;195: 171–8. 10.1002/path.918 11592095

[30] Minoo P, Zlobec I, Peterson M, Terracciano L, Lugli A. Characterization of rectal, proximal and distal colon cancers based on clinicopathological, molecular and protein profiles. Int J Oncol. 2010;37: 707–18. 20664940 10.3892/ijo_00000720

[31] Aljada A, Mousa SA. Metformin and neoplasia: implications and indications. Pharmacol Ther. 2012;133: 108–15. 10.1016/j.pharmthera.2011.09.004 21924289

[32] Hajjar J, Habra MA, Naing A. Metformin: an old drug with new potential. Expert Opin Investig Drugs. 2013;22: 1511–7. 10.1517/13543784.2013.833604 23978196

[33] Wu L, Zhu J, Prokop LJ, Murad MH. Pharmacologic Therapy of Diabetes and Overall Cancer Risk and Mortality: A Meta-Analysis of 265 Studies. Sci Rep. 2015;15:10147. 10.1038/srep10147 PMC4467243 26076034

[34] Hosono K, Endo H, Takahashi H, Sugiyama M, Sakai E, Uchiyama T, et al Metformin suppresses colorectal aberrant crypt foci in a short-term clinical trial. Cancer Prev Res (Phila). 2010;3:1077–83. 20810669 10.1158/1940-6207.CAPR-10-0186

[35] Lega IC, Shah PS, Margel D, Beyene J, Rochon PA, Lipscombe LL. The effect of metformin on mortality following cancer among patients with diabetes. Cancer Epidemiol Biomarkers Prev. 2014;23: 1974–84. 10.1158/1055-9965.EPI-14-0327 25030683

[36] Sui X, Xu Y, Yang J, Fang Y, Lou H, et al Use of metformin alone is not associated with survival outcomes of colorectal cancer cell but AMPK activator AICAR sensitizes anticancer effect of 5-fluorouracil through AMPK activation. PLoS One. 2014;9: e97781 10.1371/journal.pone.0097781 24849329 PMC4029793

[37] Lee KM, Lee M, Lee J, Kim SW, Moon HG, Noh DY, et al Enhanced anti-tumor activity and cytotoxic effect on cancer stem cell population of metformin-butyrate compared with metformin HCl in breast cancer. Oncotarget. 2016;7:38500–38512. 10.18632/oncotarget.9522 27223262 PMC5122406

[38] Graham ML, Janecek JL, Kittredge JA, Hering BJ, Schuurman HJ. The streptozotocin-induced diabetic nude mouse model: differences between animals from different sources. Comp Med. 2011;61: 356–60. 22330251 PMC3155402

[39] Lee J, Giovannucci E, Jeon JY. Diabetes and mortality in patients with prostate cancer: a meta-analysis. Springerplus. 2016;5: 1548 10.1186/s40064-016-3233-y 27652121 PMC5021649

