# Supplementary figures and images for: Colon cancer modulation by a diabetic environment: A single institutional experience
Source: PLoS One. 2017 Mar 2;12(3):e0172300. doi: 10.1371/journal.pone.0172300 (PMC5333811; doi:10.1371/journal.pone.0172300)

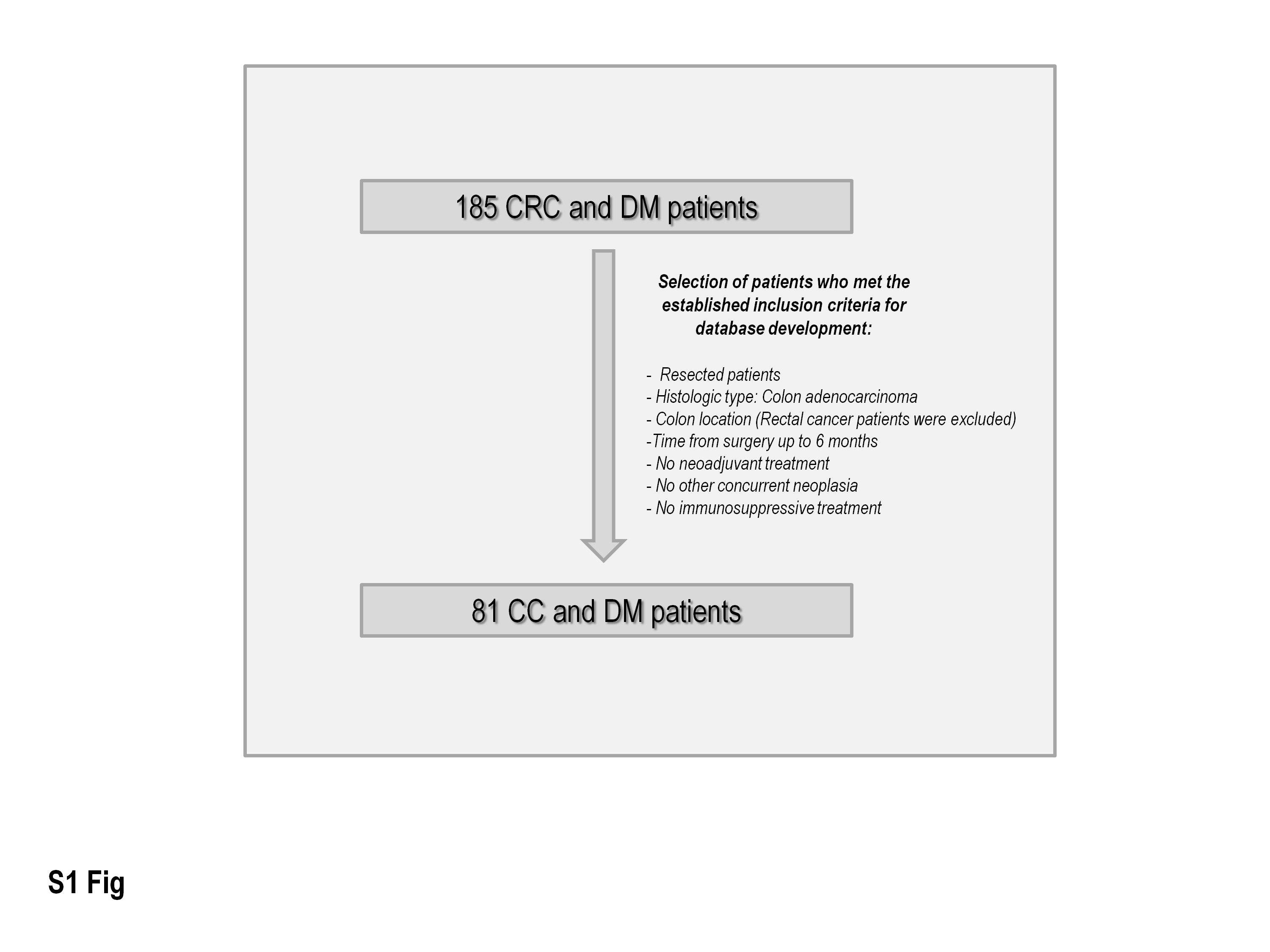

Supplement: S1 Fig — (TIF) [file pone.0172300.s001.tif]
